# Adopting the Situation in School Questionnaire to Examine Physical Education Teachers’ Motivating and Demotivating Styles Using a Circumplex Approach

**DOI:** 10.3390/ijerph18147342

**Published:** 2021-07-09

**Authors:** Géraldine Escriva-Boulley, Emma Guillet-Descas, Nathalie Aelterman, Maarten Vansteenkiste, Nele Van Doren, Vanessa Lentillon-Kaestner, Leen Haerens

**Affiliations:** 1 LISEC Laboratory, Haute Alsace University, 68093 Mulhouse, France; 2 Laboratory of Vulnerabilities and Innovation in Sport (L-VIS UR 7428), University of Claude Bernard Lyon 1, 69100 Villeurbanne, France; emma.guillet@univ-lyon1.fr; 3 Impetus Academy Inc., 9910 Knesselare, Belgium; nathalie.aelterman@impetus.academy; 4 Department of Personality, Social and Developmental Psychology, Faculty of Psychology and Educational Sciences, Ghent University, 9000 Ghent, Belgium; Maarten.Vansteenkiste@UGent.be; 5 Department of Movement and Sports Sciences, Faculty of Medicine and Health Sciences, Ghent University, 9000 Ghent, Belgium; Nele.VanDoren@UGent.be; 6 Teaching and Research Unit in Physical Education and Sport (UER-EPS), University of Teacher Education, State of Vaud (HEP Vaud), CH1014 Lausanne, Switzerland; vanessa.lentillon@hepl.ch

**Keywords:** autonomy support, structure, control, motivation to teach, need support, self-determination theory

## Abstract

Grounded in SDT, several studies have highlighted the role of teachers’ motivating and demotivating styles for students’ motivation, learning, and physical activity in physical education (PE). However, most of these studies focused on a restricted number of motivating strategies (e.g., offering choice) or dimensions (e.g., autonomy support). Recently, researchers have developed the Situations-in-School (i.e., SIS-Education) questionnaire, which allows one to gain a more integrative and fine-grained insight into teachers’ engagement in autonomy-support, structure, control, and chaos through a circular structure (i.e., a circumplex). Although teaching in PE resembles teaching in academic courses in many ways, some of the items of the original situation-based questionnaire (e.g., regarding homework) are irrelevant to the PE context. In the present study, we therefore sought to develop a modified, PE-friendly version of this earlier validated SIS-questionnaire—the SIS-PE. Findings in a sample of Belgian (N = 136) and French (N = 259) PE teachers, examined together and as independent samples, showed that the variation in PE teachers’ motivating styles in this adapted version is also best captured by a circumplex structure, with four overarching styles and eight subareas differing in their level of need support and directiveness. The SIS-PE possesses excellent convergent and concurrent validity. With the adaptations being successful, great opportunities for future research on PE teachers (de-)motivating styles are created.

## 1. Introduction

Through their motivating style, i.e., the interpersonal sentiment and behaviors teachers use to motivate their students [1], physical education (PE) teachers play a major role in fostering positive experiences in PE. Yet, teachers can also adopt a demotivating style, hereby inducting negative student experiences. According to Self-Determination Theory (SDT; [2]) teachers’ motivating style is characterized by the provision of autonomy-support [3], structure [4] and involvement [5]. By contrast, teachers demotivating style is described as controlling [6], chaotic [7] and cold [8]. A characteristic of the current SDT-based literature on teachers’ motivating or demotivating style in PE is that, in most studies, only one (e.g., autonomy-support) or two dimensions (e.g., autonomy-support and control) of teachers’ styles are examined, particularly with a chaotic style being neglected. The few studies that examined a more extensive number of dimensions tended to make use of a more global sum score, combining several motivating (i.e., autonomy-support, structure, involvement) or demotivating dimensions (i.e., control, chaos, coldness), and did not examine the strategies behind these dimensions (e.g., [9,10]). The aim of the current study, therefore, is to develop and validate a questionnaire (see Supplementary Table S1) that allows measurement of several dimensions of PE teachers (de-)motivating styles simultaneously, hereby taking a more integrative approach. 

**Figure 1:**
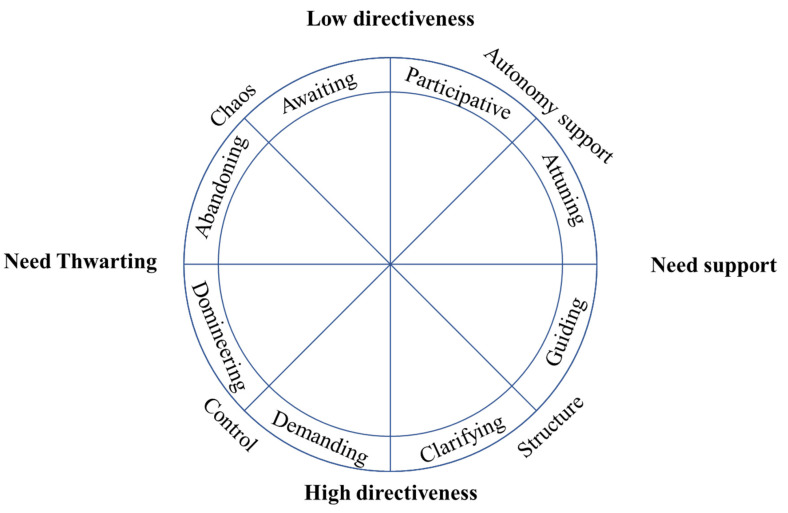
Graphical representation of the circumplex model (Aelterman et al., 2019).

Aelterman and colleagues [11] recently developed the Situations-in-School (i.e., SIS-Education) questionnaire, a situation-based questionnaire, that allows one to provide an integrative insight into teachers’ (de-)motivating styles (see Figure 1) as it measures autonomy support, structure, control, and chaos simultaneously. This questionnaire was originally developed to measure secondary school teachers’ (de-)motivating styles and was later adapted to the context of sports [12] and teaching in higher education [13]. To date, an equivalent questionnaire to measure PE teachers’ (de-)motivating styles is not available. Although teaching in PE resembles teaching in academic courses and coaching in sports in a number of ways, there are also some important differences (e.g., no homework is given, students are not sitting behind desks), making some of the situations and items of the original situation-based questionnaire irrelevant to the PE context. As such, the first aim of the present study is to develop and validate a modified version of the SIS-Education questionnaire, the SIS-PE [11]. A second aim of the current study is to examine whether the scales of the newly developed SIS-PE questionnaire relate in meaningful and theoretically expected ways to teachers’ motivation to teach. 

### 1.1. Need-Supportive Teaching as Presented in the Circumplex Model: Autonomy Support and Structure

Autonomy-supportive PE teachers attempt to identify, develop, and nurture students’ interests [3] by listening to what students have to say, taking into account their preferences and explaining the meaning of assigned exercises. Most studies investigating PE teachers’ motivating styles were centered on the provision of autonomy support [14]. Cross-sectional [15,16,17] and intervention-based studies [18,19,20,21,22,23]; which showed that teachers can be successfully trained to adopt autonomy-supportive strategies] have found that students’ perceptions of teacher autonomy support are positively related to a range of outcomes, including greater satisfaction of the three basic psychological needs, i.e., needs for autonomy (i.e., feeling a sense of initiative and self-endorsement), competence (i.e., feeling effective) and relatedness (i.e., feeling cared for) (e.g., [17,20]); students’ engagement (e.g., [15,19]); prosocial behavior [17]; and skills development (e.g., [16,18]). Furthermore, intervention studies also showed that teachers can be successfully trained to adopt an autonomy-supportive style, to the benefit of their students but also themselves. Trained teachers report increased teaching motivation, improved teaching skills and higher teaching well-being compared to those who did not follow a training [21].

When teachers adopt a structuring style, they aim to facilitate students’ competence development [2]. Structure denotes both the amount and clarity of information that PE teachers provide to students about their expectations as well as the necessary guidance “how to” effectively achieve desired outcomes [8,24]. Structuring teaching allows students to experience greater competence need satisfaction [4,25], positive affect [26], and to apply more effective learning strategies [26]. Just as with autonomy support, intervention research with PE teachers revealed that teachers can learn to adopt a more structuring style [22,27,28].

### 1.2. Need-Thwarting Teaching as Presented in the Circumplex Model: Control and Chaos

If the need-supportive teaching styles (i.e., autonomy support and structure) represent the bright side, the need-thwarting teaching strategies (i.e., control and chaos) represent the dark side of teachers’ motivating style [16,29,30]. Need-thwarting teaching strategies, specifically controlling strategies, have increasingly received attention in the PE context. Controlling teaching involves giving instruction and relating to students in a way that pressures them to think, feel, and behave in teacher-prescribed ways [1]. In PE, controlling teaching has been found to be negatively associated with psychological need satisfaction, autonomous motivation [31], prosocial behavior [20] and students’ engagement [15]. In contrast, it was positively related to need frustration and controlled forms of motivation [6,31].

Teachers who adopt a chaotic teaching style are confusing, contradictory, and unpredictable, thereby preventing students from understanding what is expected from them and how to live up to and achieve these expectations [5,32]. In the SDT literature, also outside the context of PE, chaotic strategies have been largely neglected. Only a few studies examined aspects of teachers’ chaotic teaching in PE [7,9] and showed inconclusive results, partially because it appeared difficult to create sound measures.

### 1.3. An Integrative Approach to Measuring PE-Teachers (de-)Motivating Styles

A more integrative and fine-grained insight into these two motivating (i.e., autonomy support, structure) and two demotivating (i.e., control, chaos) styles was achieved through the application of Multidimensional Scaling (i.e., MDS) analyses on a newly developed questionnaire. Across seven samples, involving both Belgian students and teachers in secondary education, a circular structure was obtained which can be found in Figure 1. The horizontal axis (i.e., *x*-axis) of the circumplex model represents the degree to which the teacher supports relative to thwarts students’ psychological needs with autonomy support and structure yielding a positive loading and control and chaos yielding a negative loading. The vertical axis reflects the extent to which the teacher is highly directive and is taking the lead or whether, instead, students are taking more of the lead, with structure, control positively loading, and chaos and autonomy support negatively loading on this dimension of directiveness.

The circumplex provides a better insight into the exact location of different (de)motivating styles in relation to each other as well as a more nuanced insight into the differentiation within each style. The circumplex makes clear that the four dimensions (i.e., autonomy support, structure, control, and chaos) can be further partitioned into two subareas each (see Figure 1). Autonomy-supportive teachers adopt *participative* and *attuning* teaching strategies (Table 1). When participative, teachers allow for students to have a say and to participate in a joint decision process. For instance, offering choice, asking for students’ input and welcoming their suggestions represent participative strategies [11,27,33]. When being attuning, teachers adopt several teaching strategies such as nurturing students’ personal interests, acknowledging their negative affect and resistance, and offering a meaningful rationale [3,11,15]. Structuring teaching strategies can be divided into *clarifying* and *guiding* teaching strategies (Table 1). When clarifying, teachers set clear expectations and goals, and scaffold students’ progress [32,34]. When guiding, teachers express confidence in the students’ capacity, they encourage their students in a constructive way, and they offer adjusted and helpful information and suggestions (e.g., feedback) to support students’ progress [4,25,32]. When controlling, teachers can adopt *demanding* and *domineering* teaching strategies (Table 1). Teachers who use demanding strategies emphasize students’ duties and responsibilities, thereby using forceful language, threats of sanctions, or the contingent use of rewards (e.g., [11,35,36]). Domineering teaching strategies involve the use of power-assertive practices such as excessive personal control, intimidation, guilt-induction, and shaming (e.g., [11,37]). Teachers who use domineering strategies are considered highly intrusive and manipulative as the target of the domineering strategies involves the student as a person instead of the student’s behavior. Chaos can be divided into *abandoning* and *awaiting* teaching strategies (Table 1). Teachers who adopt abandoning teaching strategies leave their students to their own devices, because they feel unable or because after they intervened several times, they have given up providing their students with information or help they needed. Awaiting teaching strategies can be related to a “laisser-faire” attitude: teachers fail to provide clear expectations, guidelines, or rules, and they expect students to be independent and take initiative themselves. Thus, they wait to see what will happen.

The subdivision in zones allows for a more fine-grained insight into the complex dynamics of teaching. Prior research among teachers [11,13] and sport coaches [12] revealed that two adjacent subareas are more positively correlated (being indicative of their compatible nature), and correlations with other areas becoming weaker and even negative (being indicative of their more conflictual nature) when moving along the circular structure. This suggests that a graded rather than categorical approach to teachers’ (de)motivating styles need to be considered [38]. Not all motivating styles nurture students’ psychological needs to the same extent, neither do all demotivating styles yield a similar need-thwarting effect. Also, the circumplex highlights the pitfalls associated with the incorrect application of motivating styles. If choice and participation are not offered in a motivating way, they may be perceived as more awaiting. Similarly, the provision of expectations and monitoring may shift to a more demotivating side if presented in a controlling way. Such statements are confirmed through this circular approach as, respectively, the participative subarea lies next to the awaiting subarea, and the clarifying subarea lies next to the demanding subarea.

### 1.4. Antecedent of Teachers’(de-)Motivating Style

According to the motivational sequence of SDT (e.g., [39]), one possible proximal and relevant antecedent of need-supportive teaching strategies in PE includes teachers’ own motivation. SDT conceptualizes motivation in terms of a continuum of self-determination ranging from autonomous motivation to controlled motivation [2]. Autonomous motivation, the most self-determined form of motivation, is characterized by a sense of volition and approbation towards specific activities and refers to two types of regulations; intrinsic motivation (i.e., the inherent pleasure and interest derived from the activity) and identified regulation (i.e., the recognition of the value and importance of an activity). Controlled motivation is characterized by feelings of pressure and involves introjected regulation (i.e., internal pressure such as a desire to avoid feelings of guilt and feeling better about oneself) and external regulation (i.e., external pressure such as a desire to obtain rewards or to avoid criticism). Finally, this continuum also includes amotivation, which represents the absence of motivation or the lack of intention to engage in a task because success is not expected despite the efforts consented, or because one does not see the point of it [2,40].

Authors have considered these motivational constructs as antecedents of teachers’ (de-)motivating style. Past studies have shown that autonomously motivated teachers use more autonomy support (e.g., [41,42]), more structure and/or involvement [43] and rely less on a controlling style [37]. In contrast, teachers who feel controlled motivated or amotivated tend to rely less on autonomy support and structure and/or interpersonal involvement [39,43], while they rely more on a demotivating style (see [44] for an overview of antecedents of controlling style, [7]). None of these studies investigated teachers’ motivation (autonomous, controlled and amotivation) in relation to both need-supportive and need-thwarting styles.

### 1.5. The Present Study

In the present study, we sought to identify the circumplex model of PE teachers’ need-supportive (i.e., autonomy support and structure) and need-thwarting (i.e., control and chaos) teaching strategies hereby using the PE-version of the earlier developed and validated SIS-questionnaire [11] in two samples of French and Belgian PE teachers. We choose to include a Belgian and French sample to provide confidence that the scale will work in different languages and for different contexts. Based on European reports [45,46], we can conclude that the way PE is taught in Belgium and France has similarities and differences. For instance, the proportion of teaching time allocated to PE is higher in France. Consistent with previous work in samples of secondary school teachers [11,13] and sport coaches [12], we expected that the (de-)motivating styles represented by autonomy-support, control, structure, and chaos would be organized along two dimensions: a horizontal dimension indicating the degree of need-support, relative to need-thwarting, and a vertical dimension indicating the level of directiveness (H1). We also expected that each of the four (de-)motivating styles could be segmented into two distinct subareas, representing the eight subareas identified in previous research (participative, attuning, guiding, clarifying, demanding, domineering, abandoning, awaiting) (H2). To gain confidence in the stability of this model, we examined whether a similar structure would emerge within PE teachers from two different countries, namely Belgium and France (H3). Further, congruent with the circular structure of the data, we expected to find a sinusoid pattern of correlations (proving the internal validity of the model). That is, we expected the correlations between two adjacent subareas to be positive, while the correlations are expected to become weaker and even negative as one moves along the circular structure of the model (H4). We also aimed to provide evidence for the convergent validity (H5) and concurrent validity (H6) of the SIS-PE. Similar to Aelterman et al.’s [11] study, we examined the relation between the four (de-)motivating styles and the eight subareas, on the one hand, and the dimensions of commonly used (de-)motivating style measures (Teacher As Social Context Questionnaire, TASCQ [47]; Psychologically Controlling Teaching, PCT [37]) on the other hand. It was hypothesized that the four (de-)motivating styles and the eight subareas would correlate most strongly with the corresponding validation measures (H5). We investigated the concurrent validity of the SIS-PE by examining antecedents of PE teachers’ (de-)motivating styles. Based on SDT motivational sequence and prior research [39], we hypothesized that autonomous motivation to teach would be positively related to the need-supportive strategies and negatively to the need-thwarting strategies. In contrast, both controlled motivation and amotivation to teach would yield a reversed pattern of relation (H6). For the latter analysis, we controlled for gender and teaching experiences as these characteristics can affect the pedagogy adopted [39].

## 2. Materials and Methods

### 2.1. Sample and Procedure

A convenience sample of PE teachers were contacted by email and online posts to fill out an online questionnaire. The email/online post explained the purpose of the study and contained a letter of presentation and a weblink to the online questionnaires. A total 259 French PE teachers and 86 Belgian PE teachers filled out a list of demographic questions as well as the SIS-PE questionnaire. The French sample (138 men, 53%) consisted of teachers who were on average 45.36 years old (*SD* = 9.73, range = 23–64) and had 20.94 years of teaching experience (*SD* = 10.29, range = 1–42). The Belgian teachers (41 men, 48%) were on average 38.35 years old (*SD* = 11.64, range = 20–61) and had 15.17 years of teaching experience (*SD* = 10.97, range = 0–39). An additional sample of 50 Belgian PE teachers filled out the SIS-PE questionnaire through their participation in an online learning environment designed to optimize their motivating style. From these teachers, no demographic data were obtained. This results in a total sample of 136 Belgian teachers who filled out the SIS-PE questionnaire.

A subsample of 69 Belgian PE teachers (31 men) filled out an optional questionnaire to determine convergent validity (see Measures). These 69 teachers were on average 38.75 years old (*SD* = 11.75, range = 21–61) and had 15.71 years of experience (*SD* = 11.38, range = 0–39). To determine concurrent validity, all teachers in the French sample filled out an additional questionnaire measuring their motivation to teach (see Measures). All participating teachers gave online consent after being informed that their participation was voluntary, that the collected data would be treated confidentially, and that they could withdraw from the study at any time, without having to give a reason and without any consequences. The study protocol was approved by the ethical guidelines of Lyon 1 University (French sample) and the Committee for Medical Ethics affiliated to UZ Ghent (Belgian sample).

### 2.2. Measures

#### 2.2.1. SIS-PE Questionnaire

The SIS questionnaire [11] presents a variety of 12 concrete situations, which teachers get confronted with on a daily basis. For every situation presented, there are four different reactions displayed, with each reaction corresponding to one of the four (de-)motivating styles (autonomy support, control, structure, and chaos). Teachers are asked to indicate to what extent each reaction reflects their way of teaching on a 7-point Likert scale ranging from one (does not describe me at all) to seven (describes me extremely well). The original SIS questionnaire has a good internal consistency with a Cronbach’s alpha ranging between 0.78 and 0.82 for the (de-)motivating styles and between 0.73 and 0.82 for the subareas [11]. The test-retest reliability of the original SIS is high with correlations between 0.48 and 0.80 [11], and both the convergent and concurrent validity (e.g., teaching motivation and burnout) of this questionnaire have also been proven. The original SIS-situations and items are presented in Table 2 (see supplementary material for translation in Dutch and French).

The PE-version of the SIS consists of 12 situations and 48 items of which 11 situations and 38 items are almost identical to the original SIS [11]. For some situations and items, a few words were slightly changed to suit the PE context better (see Table 2). For example, the situation “6”: ‘You present a difficult lesson that requires a lot of effort from the students. In doing so, you…’, was changed into: ‘You are presenting a difficult exercise that requires a lot of effort for the students. In doing so, you…’. One situation (i.e., situation 12, regarding homework) and 10 items that are indicated in bold in Table 2 were substantially changed because these were rather irrelevant in the context of PE. To illustrate, the item ‘Pound the desk and say loudly: “Now it is time to pay attention! “’was, changed into, ‘whistle and say loud and clear, “Now let’s focus and get busy.”’. Among these 10 items three were “autonomy-supportive” items (two participative and one attuning), three were “structure” items (two guiding and one clarifying), two were “control” items (one domineering and one demanding) and two were “chaos” items (abandoning). Ultimately, the SIS-PE questionnaire consists of 12 autonomy-supportive items (four participative, eight attuning), 12 structuring items (seven guiding, five clarifying), 12 controlling items (seven demanding, five domineering), and 12 chaos items (eight abandoning, four awaiting). The SIS-PE questionnaire was first developed in Dutch and was then translated in French. To do so, back-and forward translation procedures were used [48].

#### 2.2.2. Convergent Validation Measures

Three subscales of the short version of the TASCQ were used to assess autonomy support (six items; e.g., “I listened to the ideas of the students of this class”), structure (five items; e.g., “I clarified my expectations to the students of this class”) and involvement (six items; e.g., “The students of this class are easy to like”). With McDonald’s omega of 0.90, 0.89, and 0.78 for autonomy support, structure, and involvement, respectively, internal consistencies were moderate to satisfactory. The PCT included nine items (e.g., “I’m less friendly to my students if they do not see things my way”) and showed a satisfactory internal consistency (ω = 0.78). For both questionnaires, items were rated on a 5-point Likert scale ranging between 1 (completely disagree) and 5 (completely agree).

#### 2.2.3. Concurrent Validation Measure

French PE teachers’ motivation was measured using the French version of Work Tasks Motivation Scale for Teachers (WTMST; [49]). The stem “I teach PE…” was followed by 15 possible reasons (three items for each dimension) which represent teachers’ intrinsic motivation (e.g., “…because I find it interesting to do”), identified regulation (e.g., “… because I find it important for the academic success of my students”), introjected regulation (e.g., “… because I would feel guilty not doing it”), external regulation (e.g., “… because I’m paid to do it”) and amotivation (e.g., “… I don’t know, I don’t always see the relevance of teaching PE”). Teachers rated each item on 7-point scale ranging from 1 (does not correspond at all) to 7 (corresponds completely). The composite scores for autonomous (intrinsic motivation and identified regulation) and controlled (introjected and external regulations) motivations were calculated. Omega for autonomous motivation, controlled motivation and amotivation were satisfactory with ω = 0.87, ω = 0.85, and ω = 0.65, respectively.

### 2.3. Plan of Analysis

To examine the dimensional structure of the SIS-PE items, that is whether the items could be organized along two dimensions (H1) and whether the four (de-)motivating styles could be segmented into two distinct teaching strategies (H2), Multidimensional Scaling (MDS; [50]) was conducted using the Proxscal procedure in SPSS 21. Specifically, MDS provides a graphical representation of (dis)similarities between elements (i.e., items) in the form of distances between points in a geometric space. That is, items that are strongly positively correlated will be represented closely to each other in the geographical space (i.e., responses are compatible), strongly negatively correlated items will be displayed in the opposite direction (responses are conflictual in nature) and items that are poorly correlated will be represented by larger distances. This analysis will allow to examine whether the items are located in their expected area. MDS was performed on Belgian data and French data together and separately in order to obtain a country-specific configuration. Differences between the SIS-PE version and the original SIS-questionnaire were inspected. To investigate the stability of the dimensional structure across countries (H3), we subjected the obtained Belgian and French configurations to Generalized Procrustes Analysis (GPA; [50]). GPA calculates configurations from different samples in such a way that they correspond as closely as possible, without affecting the relative distances between items within each configuration. Further, to test the internal validity of the model, through correlational analyses, we examined the pattern of correlates between the subareas (H4). To investigate the convergent validity of the SIS-PE, we examined whether the dimensions and subareas correlated with the convergent validation measures (i.e., TASCQ and PCT) in a meaningful way (H5). Finally, the concurrent validity was determined by examining the antecedents of teachers’ (de)motivating style (H6). We performed correlation analysis and hierarchical regressions analyses with teachers’ motivation. These variables were examined independently, and analyses were controlled for demographic and professional variables.

## 3. Results

### 3.1. Are the Items Organized along Two Dimensions (H1)?

To investigate whether the variety of assessed teaching strategies were organized along two dimensions, we evaluated several configurations ranging from a one-dimensional up to a six-dimensional solution produced by non-metric MDS. We opted for a two-dimensional instead of single-dimensional solution. That is because, in the total sample, the normalized raw stress declined from 0.099 for the one-dimensional representation to 0.025 for the two-dimensional representation, while only a small decline to 0.015 observed when retaining three dimensions in the whole sample. Similar findings were obtained for the two samples separately. In the Belgian sample, the normalized raw stress declined from 0.122 for the one-dimensional representation to 0.035 for the two-dimensional representation, while only a small decline to 0.020 observed when retaining three dimensions. In the French sample, the normalized raw stress declined from 0.105 for the one-dimensional representation to 0.034 for the two-dimensional representation, while only a small decline to 0.021 observed when retaining three dimensions. When withholding two dimensions, 97% of the distances were represented in the model for all three samples.

**Figure 2:**
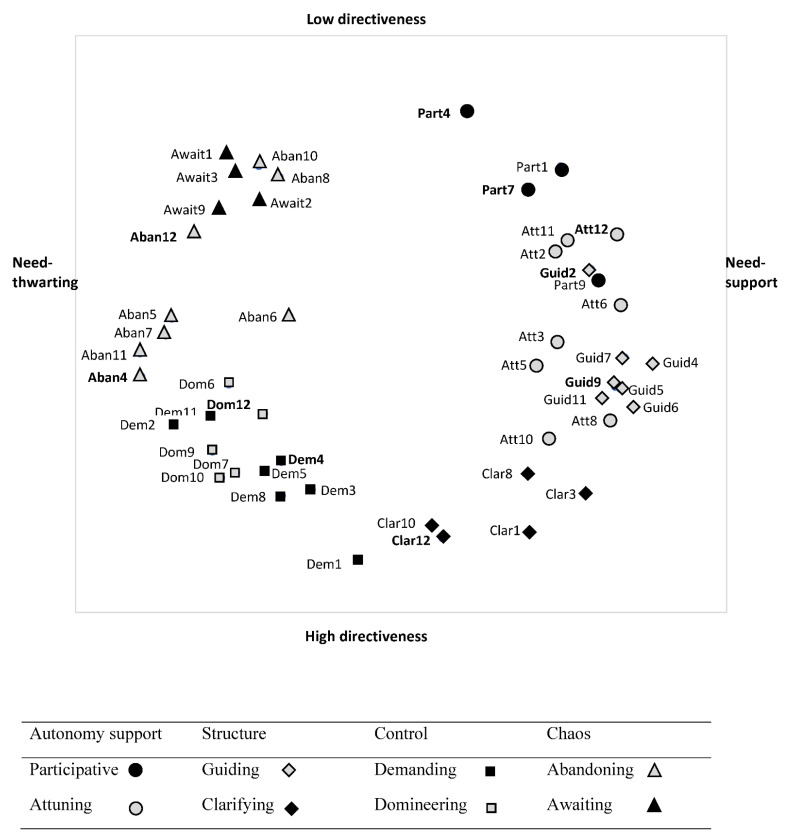
Two-dimensional representation of the simulations-in-schools–physical education (SIS-PE) items in Belgium and France.

In line with previous studies [11,12,13], the *x*-axis of the two-dimensional representation (i.e., the first dimension) reflected a continuum from need-supportive to need-thwarting teaching strategies and, the y-axis (i.e., the second dimension) could be interpreted as the degree of PE teacher directiveness. In the Belgian sample, four broader quadrants could be distinguished, with most structure items being situated in the lower right quadrant, most autonomy-supportive items in the upper right quadrant, the chaos items in the upper left quadrant, and the control items in the lower left quadrant. This global structure was somewhat less clear when only taking into account the French sample and when combining the two samples (Figure 2), because the attuning (as part of autonomy support) and guiding (as part of structure) items somewhat collapsed in between the participative and clarifying items. The upper left quarter did largely present a chaotic style and the lower left quarter represented a controlling style. Given that the global structure was largely replicated, we relied on the definition of the items as depicted in the original SIS-education ([11]; see Table 2) to calculate sum scores the four (de)motivating dimensions of autonomy support, structure, control, and chaos. The scales showed moderate to good internal consistencies with omega values ranging between 0.71 and 0.86 (Table 3, Table 4 and Table 5).

### 3.2. Are the Four (de-)Motivating Styles Segmented into Two Distinct Teaching Strategies (H2)?

**Figure 3:**
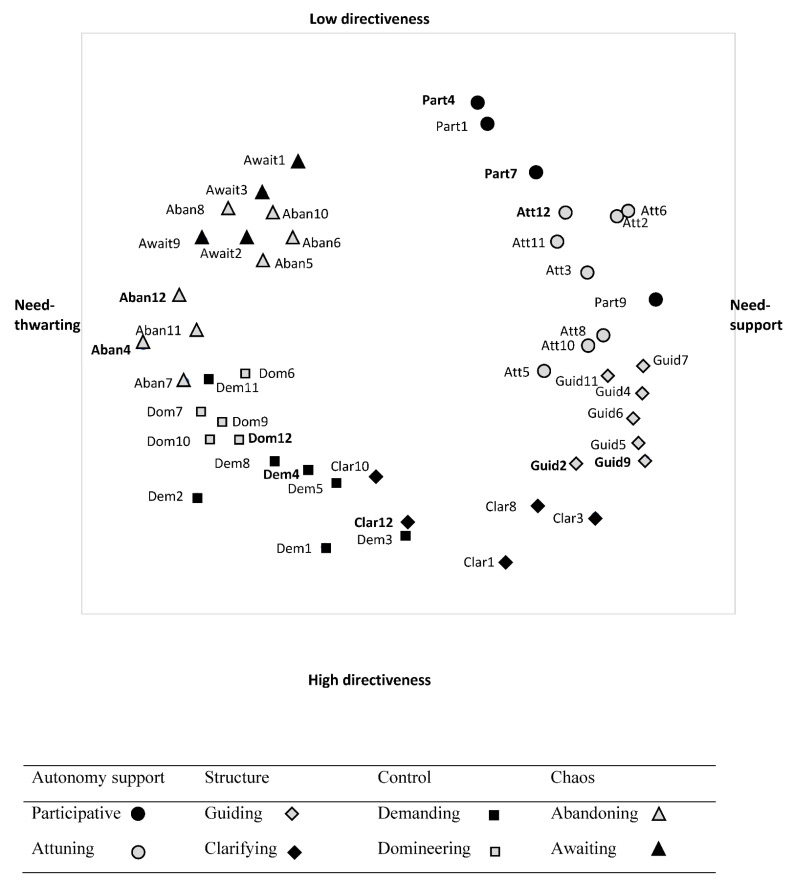
Two-dimensional representation of the SIS-PE items in Belgium.

**Figure 4:**
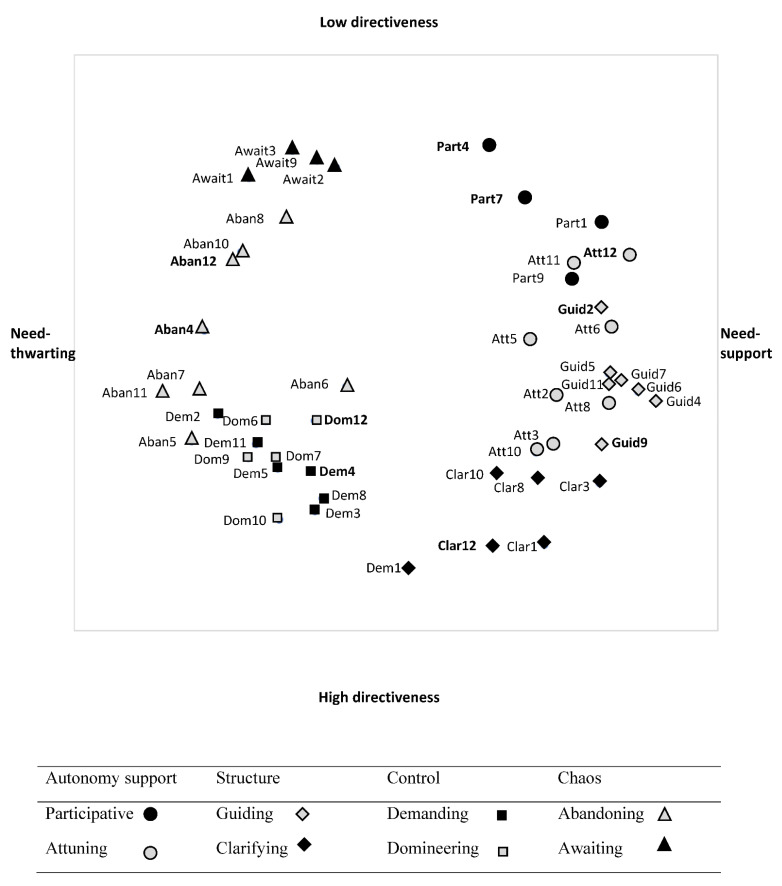
Two-dimensional representation of the SIS-PE items in France.

The eight subareas as identified by Aelterman et al., [11] were most clearly identified in the Belgian sample, while this was somewhat less clear in the total sample and in the French sample because the guiding and attuning, as well as the domineering and demanding subareas somewhat collapsed. Note that all 10 newly formulated items (i.e., part4, part7, att12, guid2, guid9, clar12, dem4, dom12, aban4, and aban12) fell in their intended area in both the Belgian (Figure 3) and the French sample (Figure 4).

Relying on the definitions of the scales in the original SIS-Education (Table 2), we then checked the structure of the subareas performing a series of CFAs on the items of two adjacent approaches. Precisely, we compared a two factor versus a single-factor solution (see [12] for similar analyses). Except for two pairs of adjacent areas (demanding/domineering and participative/attuning) in the French sample (Δχ^2^ = 1.89 and Δχ^2^ = 0.04, respectively), and one pair (participative/attuning) in the total sample (Δχ^2^ = 1.63), results of χ^2^ change tests showed that a two-factor solution was more suitable than one factor solution. The fits improved for each test from a one-factor to a two-factor solution (6.41 < Δχ^2^ < 131.68, *p* = 0.05). Moreover, reliability analyses showed that, except for the participative subarea (ω = 0.45 in the total sample; ω = 0.44 in Belgian sample and ω = 0.41 in French sample), internal consistencies for the all the subareas were acceptable to good, varying between 0.69 and 0.82 (see Table 3, Table 4 and Table 5).

### 3.3. Stability of the Circumplex Structure across Countries (H3)

Results from the MDS analyses showed that a two-dimensional circumplex structure emerged from both the Belgian and French data, and that the graphical representations of the SIS-PE items appeared similar between both countries. We performed GPA to the sample-specific configurations in order to examine whether the obtained solution is indeed similar across countries (H3). In total, 96% of the (squared) distances in the two sample-specific configurations could be represented in a single consensus configuration meaning that only 4% of the squared distances was lost by representing the two sample-specific configurations by a single centroid configuration. Furthermore, we correlated the coordinates of the items on both dimensions in the consensus configuration with the coordinates of the items in the separate Belgian and French configurations. The need-support (versus need-thwarting) dimension of the consensus configuration was significantly and positively correlated 0.99 and 0.97 with the corresponding dimension in Belgian (*r* = 0.99) and French (*r* = 0.99) configurations. Next, the directiveness dimension yielded a significant and positive correlation with the corresponding dimension in Belgian (*r* = 0.95) and French (*r* = 0.95) configurations. Additionally, we correlated the coordinates of the items on both dimensions in the consensus configuration with the coordinates of both Belgian and French configurations. The need-support dimension in Belgian and French were significantly and positively correlated (*r* = 0.95) and similar results were found for the directiveness dimension (*r* = 0.81). Together, these results indicate that the two-dimensional structure is stable across countries, which further supports the validity of the configuration.

### 3.4. Correlational Pattern (H4)

As presented in Table 4 for the Belgian sample and Table 5 for the French sample, autonomy support and structure, on the one hand, and control and chaos, on the other hand, were positively correlated to each other. By contrast, except for the non-significant correlation between structure and control in the French sample, autonomy support, and structure were negatively correlated to control and chaos. Further, evidence of an ordered pattern of correlations between the eight subareas was found. Except for the participative and awaiting subareas, which are unrelated in all analyses, all adjacent subareas were positively correlated, and these correlations were among the strongest. It is worth noticing that, among these strongest correlation correlations between clarifying and demanding are among the lowest. The strength of the correlations decreased and even became negative when moving along the circumplex to subareas situated at the opposite side (H4). For instance, in the Belgian sample, the attuning subarea was (1) positively correlated to the participative and guiding subareas (strongest positive correlations), (2) positively correlated to the clarifying subarea and unrelated to the awaiting subarea and (3) while being negatively correlated to the domineering, abandoning and demanding subareas with the negative correlation with domineering (exact opposite) being the strongest. Other subareas followed a similar pattern of correlations.

### 3.5. Convergent Validity (H5)

Correlation analysis of the four dimensions and eight subareas with their corresponding convergent validation measures are presented in Table 4. Specifically, autonomy support, structure, and control in SIS-PE were most strongly and positively correlated to, respectively, autonomy support, structure from the TASCQ and control from the PCT providing support for its convergent validity (H5). Additionally, the participative and the attuning subdimensions were both positively and strongly correlated to autonomy support from the TASCQ. Both the guiding and clarifying subdimensions of structure were positively correlated to structure from TASCQ. The demanding and domineering subdimensions were positively correlated to control (PCT). It is also interesting to note that both autonomy-supportive subareas as well as the guiding subarea positively correlated with teachers’ involvement as measured by the TASCQ.

### 3.6. Concurrent Validity (H6)

Table 5 presented correlations between the variables without controlling for gender and years of experience. Results from regression analyses showed that, after controlling for teachers’ gender and years of teaching experience, autonomous motivation was associated with the provision of autonomy support and structure, and all the corresponding subareas. Controlled motivation was predictive of control, and three out of four need-thwarting subareas (i.e., demanding, domineering, and abandoning). Amotivation positively related to control and chaos, and particularly the domineering and abandoning subareas. Regarding PE teachers’ gender, results showed that male PE teachers are more inclined to adopt controlling or chaotic styles, which is evident in higher scores for the demanding, domineering, abandoning, and awaiting subareas. Teaching experience was positively associated with clarifying subarea and structure in general, and to awaiting subarea (marginally). (Table 6).

## 4. Discussion

Grounded in SDT, several studies have highlighted the role of teachers’ motivating and demotivating styles for students’ adaptive or maladaptive outcomes in PE [14,39]. However, most of these studies focused on a restricted number of motivating strategies (e.g., offering choice) or dimensions (e.g., autonomy support). Furthermore, teachers’ demotivating styles, particularly the chaotic dimension, were largely neglected in past research. Moreover, in most prior research, a categorical approach is taken in which teachers’ styles are considered as separate and distinct entities. To overcome these limitations, the primary purpose of the present study was to develop and test a PE-version of the SIS-Education questionnaire [11] which provides a more integrative and refined picture of teachers’ (de-)motivating styles. In essence, our results showed that the adaptations of the SIS-Education to the PE context were successful. The circular structure as identified in prior research [11] could be largely replicated in the total sample as much as in the two distinct samples of Belgian and French PE teachers, with the 10 newly formulated items falling into their intended areas. Results also confirmed the convergent and concurrent validity of the scales derived from the SIS-PE questionnaire. Overall, it can be concluded that convincing evidence was provided for the validity of the SIS-PE to measure PE teachers’ motivating and demotivating styles.

### 4.1. Does the Circular Structure of the SIS-PE Questionnaire Match the Circular Structure of the SIS-Education?

In line with previous work in the contexts of education [11,13]) and sport [12], MDS analysis showed that PE teachers’ (de)motivating styles could be represented graphically by a two-dimensional circular pattern (see Figure 1). As hypothesized, the *x*-axis represented the degree to which teachers are need-supportive, relative to need-thwarting, with most autonomy-supportive and structuring items being positioned on the need-supportive side and most controlling and chaotic teaching items need-thwarting side in both samples. The y-axis represented teachers’ degree of directiveness, that is whether teachers take charge in the classroom, or leave the opportunity to students to take initiative. As expected, most controlling or structuring teaching strategies were positioned at the high directiveness side, while most autonomy-supportive and chaotic were positioned at the low directiveness side. In addition to this integrated picture, our results provided further support for the refined division into eight subareas as identified in prior research [11,12,13]. Specifically, each dimension could be partitioned into two subareas and this circular structure appeared stable across both countries.

Yet, we did find one exception. Part of the attuning and guiding subareas collapsed, particularly in the French sample and when combining both samples, but also in the Belgian sample, these items somewhat grouped together. This finding is interesting and in line with findings with the SIS-Sport [12]. Apparently, in PE and sports other than in academic subjects, attuning (e.g., providing for enjoyable exercises) and guiding (e.g., providing hints and feedback) strategies more strongly co-occur. Inspection of the graphical presentations further revealed that some of the attuning items were also more clearly differentiated from the guiding approach in all three samples, leaning more closely to a participative approach. These items referred to effectively listening to what students have to say to allow them to express their opinions and feelings (att11, att12).

Another interesting finding emerged when inspecting the position of specific items in the different subareas of the circumplex. The results regarding the “choice” items (part4 & 9) were interesting in many ways. The provision of choice is one of the key components of autonomy support [33,51,52] which is part of the participative subarea. SDT predicts that if students are allowed to choose what activity to do (i.e., option choice) or when, where, how, or with whom to do it (i.e., action choice), they will experience a greater sense of autonomy [2], particularly if the provided choices promote volition and align with students’ wishes and interests [53]. Nonetheless, debates are ongoing related to the possible pitfalls associated with offering choices ([54,55] for a meta-analysis). Reeve and colleagues [51], for instance, found action choice (i.e., choice in order, pace, with whom) to be more need-satisfying when compared to option choice (i.e., choice in the activities). Regarding option choice, studies showed that it is important that the offered options are meaningful, clearly differ from one other [53,56] and are not too numerous [57]. In the current study, there were two types of “choice” items included in the questionnaire. The first “choice” item (part9) represented the provision of action choice (i.e., choice in the difficulty level of the exercises). The second “choice” item (part4) represented option choice, referring to choice in the learning activities proposed (e.g., what activity students want to do). While the action choice item lay on the edge between the participative and attuning area, the option choice item lay on the edge between the participative and awaiting area. The inclusion of option choice may thus lead teachers to leave students too much to their own devices. Action choice instead, particularly choice in the difficulty level of the exercises, may be indicative of teachers’ intention to attune to the students’ needs. As such, the insights gained from this study may further explain, why compared to action choice, option choice could be experienced as less need-satisfying in comparison with action choice ([53,55] for a meta-analysis), an issue that warrants further examination.

### 4.2. Pattern of Correlations between the Subareas

In line with expectations [11], the eight subareas were related in an ordered or graded pattern to each other. More precisely, a sinusoid pattern of correlations appeared between subareas, with each area being positively correlated to its adjacent areas, and with correlations becoming weaker, even non-significant or negative when moving along the circumplex. The lowest correlations between adjacent strategies (even negative and/or not significant) were found for the borders between the need-supportive and need-thwarting teaching styles, i.e., between participative and awaiting. Although these subareas are lying next to each other, they still appear to be rather distinct. Similar results were found in Vermote et al.’s [13] work, showing that participative strategies covary less strongly with teachers’ awaiting strategies than with teachers’ attuning strategies. Also, in line with this previous study [13], results showed that clarifying strategies covary less strongly with teachers’ demanding strategies than with guiding strategies. Note in the graphical representations that particularly clarifying items (clar 10 & 12) that are more reactive, referring to repeating expectations about attitudes or punctuality lean more closely to the demanding subarea, while proactive items (i.e., clar8 “You communicate your expectations in terms of effort and attitude in the class”, clar3 “You set-up a clear and easy to follow organization”) lean more closely to the guiding area in both samples. According to social domain theory [58,59,60] the first set of reactive items refer social conventions (e.g., punctuality, attitude) including judgments that are subject to specific rules or orders from authority, and modifiable according to the context and thus perhaps those teachers who are more demanding may hold more value to good attitude and being punctual. The second set of proactive items refer to moral rules (e.g., effort, respect the rule), which are seen as generalizable, obligatory, inalterable across contexts and thus more easily accepted.

### 4.3. Convergent and Concurrent Validity of the SIS-PE Scales

Each of the scales of the SIS-PE correlated most strongly to its corresponding scale providing evidence for its convergent validity. Also, regarding the concurrent validity hypotheses were confirmed as results showed that autonomous motivation was related to the adoption of need supportive strategies, whereas controlled motivation and amotivation were related to need thwarting strategies. Such findings are in line with prior research [43,44]. It was also interesting to note that teachers amotivation was most strongly related to a chaotic, and more specifically to an abandoning style, which is considered the most detrimental [11]. Teachers who are amotivated do not see the meaning of teaching anymore. Clearly, this could lead teachers to fully give up on students. Regarding PE teachers’ gender and teaching experience, results showed that men used more need-thwarting strategies than women and experienced teachers used more structure (i.e., more clarifying strategies) and more awaiting strategies. These results are not in line with previous research led in the same countries. Two studies prior reported no gender differences in teachers motivating style (e.g., [9,22]), and one study reported a gender difference for structure only with male teachers providing more structure than female teachers (e.g., [7]). Regarding teaching experience, positive associations with controlling teaching have been reported in prior work [7], yet such findings were not confirmed in the current study. More research is needed to investigate how teacher gender and year of experience are related to their motivating style.

### 4.4. Limitations

The present study contains some limitations. First, although most of the scales had satisfying internal consistency, the participative scale showed poor internal consistency. Yet, in previous studies internal consistency of this scale was also among the lowest [11,12]. There is a need to improve this scale by reworking on the participative items. Second, in the present study teachers’ (de-)motivating styles were assessed based on teacher self-reports. Future studies could also include students’ reports and observational measures to examine how these correlate to teachers’ self-reports to provide further confidence in the validity of the scales. Also, the concurrent validity of the SIS-PE can be further established by including student outcomes such as their motivation, engagement, or activity levels in PE. Third, we did not examine the convergent validity for chaotic teaching strategies as, to the best of our knowledge, there are no reliable and valid measurement tool available. Fourth, we only investigated one antecedent, that is teachers’ motivation to teach. However, based on motivational sequence of SDT (e.g., [39]) and other previous studies (e.g., [61]) other antecedents such as need satisfaction/frustration and pressures perceived by teachers could be related to PE teachers’ styles. Further research is needed to investigate these antecedents. This study can set the stage for a program of research on how a fuller range of antecedents relates to each of the subareas as identified in the SIS-PE. Fifth, like the SIS-Education questionnaire, the SIS-PE vignettes did not include responses tapping into teachers’ involvement (relatedness support) and rejection (relatedness thwarting), which constitute the third (de)motivating styles presented in SDT [2,61]. Studies showed the importance of this motivating style on students’ engagement and motivation [62]. Previous studies acknowledged the need to develop additional items and explore their position in the circumplex model. In previous studies on the circumplex model it was posited that that, in line with theory relatedness supportive items would be positioned at the need-supportive side, while relatedness thwarting items would be positioned at the need-thwarting side (e.g., [11,12]). A literature review also highlighted that studies mainly focus on one component of relatedness support i.e., affection but only few investigated the other three components i.e., attunement, dedication of resources, and dependability [62]. Correlational results of the current study suggest that participative, attuning and guiding approaches most closely aligned with high levels of relatedness-support. Further investigations are needed to confirm these findings.

### 4.5. Practical Implication

With teachers’ personalized scores being graphically depicted for each of the subareas, a richer and more complete picture of teachers (de-)motivating styles can be provided. This provides great opportunities for tailored interventions. Specifically, teachers’ personalized profile can be used as a starting point for self-reflection, and to provide tailored hints and feedback to foster teachers’ personal and professional development. As to develop effective interventions, further insights into the antecedents of teachers’ (de-)motivating styles is needed. Clearly, teachers own motivation is of great importance as it meaningfully correlates with the way they interact with their students. By investigating other antecedents (e.g., pressures perceived by teachers, PE teachers’ self-efficacy) of teachers’ motivation and their (de-)motivating styles, it will become clearer how teachers’ working context can be optimized.

## 5. Conclusions

Given the major role PE teachers play in shaping students’ experiences through their (de-)motivating styles, and the consequences this has for students’ motivation and engagement in PE and in leisure time PA, future research will benefit from the more comprehensive and refined SDT-based measure of PE teachers’ (de-)motivating styles (see [14,39] for a review). The present study provides such a measure. Findings in two distinct samples, proved that the PE-version of the SIS-questionnaire [11] allows one to portray PE-teachers’ (de-)motivating styles in a circumplex structure distinguishing four dimensions and eight subareas differing in their level of need support and directiveness, which possess excellent convergent and concurrent validity.

## Figures and Tables

**Table 1:** Conceptual definitions of the four teaching styles and description of the eight identified teaching approaches (Aelterman et al., 2019).

Teaching Style	Conceptual Definition	Subarea	Description
Autonomy support	The teacher’s instructional goal and interpersonal tone of *understanding.* The teacher seeks to maximally identify and nurture students’ interests, preferences, and feelings, so that students can volitionally engage themselves in classroom learning activities.	Participative	A *participative* teacher identifies students’ personal interests by engaging in a dialogue with students and inviting them to provide input and suggestions. In addition, where possible, the teacher tries to offer (meaningful) choices in how students deal with learning activities and optimally follows their pace.
		Attuning	An *attuning* teacher nurtures students’ personal interests by trying to find ways to make the exercises more interesting and enjoyable, accepting students’ expressions of negative affect and trying to understand how students see things. The teacher allows students to work at their own pace and provides explanatory rationales that are meaningful in the eyes of students.
Structure	The teacher’s instructional goal and interpersonal tone of *guidance.* Starting from the capabilities and abilities of students, the teacher provides strategies, help and assistance, so that students feel competent to master classroom learning activities.	Guiding	A *guiding* teacher nurtures students’ progress by providing appropriate help and assistance as and when needed. The teacher goes through the steps that are necessary to complete a task, so that students can continue independently and, if necessary, can ask questions. Together with the students, the teacher constructively reflects on mistakes, so that they see for themselves what can be improved and how they can improve.
		Clarifying	A *clarifying* teacher communicates expectations to students in a clear and transparent way. The teacher offers an overview of what students can expect from the lesson and monitors students’ progress in meeting the communicated expectations.
Control	The teacher’s instructional goal and interpersonal tone of *pressure.* The teacher insists that students think, feel, and behave in a prescribed way and imposes his/her own agenda and requirements on students, irrespective of what students think.	Demanding	A *demanding* teacher requires discipline from the students by using powerful and commanding language to make clear what students have todo. The teacher points students on their duties, tolerates no participation or contradiction, and threatens with sanctions if students don’t comply
		Domineering	A *domineering* teacher exerts power to students to make them comply with his/her requests. The teacher suppresses students by inducing feelingsof guilt and shame. While a demanding teacher tries to change students’ thoughts, feelings, and behaviors into something more acceptable to theteacher, a domineering approach is characterized by a ‘personal attack’ on students.
Chaos	The teacher’s instructional goal and interpersonal tone of *laissez faire.* The teacher leaves students on their own, making it confusing for students to figure out what that they should do, how they should behave, and how they can develop their skills.	Abandoning	An *abandoning* teacher gives up on students. The teacher allows students to just do their own thing, because, eventually students have to learn to take responsibility for their own behavior.
		Awaiting	An *awaiting* teacher offers a laissez-faire learning climate where the initiative fully lies with the students. The teacher tends to wait to see howthings evolve, doesn’t plan too much and rather let things take their course.

**Table 2:** Similarities and differences between SIS and SIS-PE items.

SIS	SIS-PE	Changes
1. Classroom Rules. *You are thinking about classroom rules. So, you…*	1. Class Rules. *At the beginning of the school year, you propose operating rules. You…*	
make an announcement about your expectations and standards for being a cooperative classmate. (clar)	announce your expectations and the rules necessary for optimal cooperation. (clar1)	A few words were changed
don’t worry too much about the rules and regulations. (await)	don’t worry too much about the rules of operation and their application. You intervene when a problem arises. (await1)	A few words were changed
post your rules. Tell students they have to follow all the rules. Post the sanctions for disobeying the rules. (dem)	set out the rules that students are expected to follow. You also list the penalties for breaking them. (dem1)	A few words were changed
invite students to suggest a set of guidelines that will help them to feel comfortable in class. (part)	invite students to suggest a few rules that will help them feel comfortable during the lesson. (part1)	A few words were changed
2. Lesson Plan. *As you prepare for class, you create a lesson plan. Your top priority would be to…*	2. Lesson Plan. *In preparing for your class, you develop a lesson plan. Your priority is to...*	
communicate which learning goals you expect sudents to accomplish by the end of the lesson. (clar)	**offer challenges to the best students and provide sufficient support to exceptional students throughout their learning. (guid2)**	**Significant adaptation and modification of the strategy**
don’t plan or organize too much. The lesson will unfold itself. (await)	don’t plan the lesson too much. It will unfold on its own. (await2)	A few words were changed
offer a very interesting, highly engaging lesson. (att)	propose exercises that are pleasant, interesting, or very attractive. (att2)	A few words were changed
insist that students have to finish all their required work no exceptions, no excuses. (dem)	propose a lesson plan for all students to follow. There are no exceptions or excuses. (dem2)	A few words were changed
3. Starting Class. *The class period begins. You…*	3. Starting Class. *The class period begins. You…*	
provide a clear, step-by-step schedule and overview for the class period. (clar)	set up a clear and easy-to-follow organization. (clar3)	A few words were changed
don’t plan too much. Instead, take things as they come. (await)	start the lesson and let it unfold. (await3)	A few words were changed
insist firmly that students must learn what they are taught— your duty is to teach, their duty is to learn. (dem)	insist strongly that students must put into practice what is taught. Your duty is to teach, their duty is to learn. (dem3)	A few words were changed
are interested to know what the students know about learning topic. (att)	are interested in what students know about the learning theme. (att3)	A few words were changed
4. Motivating Students. *You would like to motivate students during class. You decide to…*	4. Motivating Students. *You would like to motivate students during class. You decide to…*	
minimize the lesson plan; let what happens happen in the lesson. (await)	**don’t take care of unmotivated students, you don’t manage to improve their motivation. (aban4)**	**Significant adaptation and modification of the strategy**
pound the desk and say loudly: “Now it is time to pay attention!” (dom)	**whistle and say loud and clear, “Now let’s focus and get busy.” (dem4)**	**Significant adaptation and modification of the strategy**
offer help and guidance. (guid)	give positive feedback, while offering help and advice when necessary. (guid4)	A few words were changed
identify what the personal benefits of the learning material are for students’ everyday life. (att)	**offer students a number of different activities that they can choose for the next cycle of education. (part4)**	**Different item**
5. Students Complain. *At a difficult point in the lesson, students begin to complain. In response, you…*	5. Students Complain. During a difficult exercise in the lesson, students start to complain. In response, you…	
accept their negative feelings as okay. Assure them that you are open to their input and suggestions. (att)	consider their frustration and explain the importance of this exercise. (att5)	A few words were changed
insist they pay attention. They must learn this material for their own good. (dem)	insist that they keep focusing. They must learn these exercises for their own good. (dem5)	A few words were changed
show and teach them a helpful strategy for how to break down the problem to solve it step-by-step. (guid)	show them the exercise step-by-step and teach them a strategy that helps them pass the exercise. (guid5)	A few words were changed
just ignore the whining and complaining. They need to learn to get over the obstacles themselves. (aban)	ignore the wailing and the complaining. They must learn to overcome obstacles on their own. (aban5)	A few words were changed
6. Needing Extra Effort. *You present a difficult lesson that requires a lot of effort from the students. In doing so, you…*	6. Needing Extra Effort. You are presenting a difficult exercise that requires a lot of effort for the students. In doing so, you…	
don’t be too concerned, as students need to figure out for themselves how much effort to put forth. (aban)	are not too worried, because students need to understand for themselves how much effort they have to put in. (aban6)	A few words were changed
try to find ways to make the lesson more interesting and enjoyable for the students. (att)	try to find new ways to make the exercise more fun and interesting for the students. (att6)	A few words were changed
insist firmly that “Now is the time for hard work!” (dom)	firmly insist that “playtime” is over and that now they must show what they are worth. (dom6)	A few words were changed
say, “Because this lesson is extra difficult, I will provide you with extra help and extra assistance, if needed.” (guid)	help the students with concrete advice on how to do the exercise successfully. (guid6)	A few words were changed
7. Anxiety Surfaces. *During a class assignment, you notice that some students are showing signs of anxiety. Sensing that anxiety, you…*	7. Anxiety Surfaces. During an exercise, you notice that some students show signs of anxiety. Sensing that anxiety, you…	
cknowledge that they look anxious and stressed. Invite them to voice their sense of unease. (att)	**talk to the students and suggest that they engage in another exercise that scares them less or not at all. (part7)**	**Significant adaptation and modification of the strategy**
insist that they must act in a more mature way. (dom)	insist that they need to move beyond this state and act in a more mature way. (dom7)	A few words were changed
break down the steps needed to handle the assigned task so that they will feel more capable of mastering it. (guid)	try to reduce their anxiety by breaking down the steps needed to complete the exercise so that they feel able to do it successfully. (guid7)	A few words were changed
don’t worry about it—let it pass on its own. (aban)	don’t have to worry about their anxiety, it will pass on by itself. (aban7)	A few words were changed
8. Student Misbehavior. *A couple of students have been rude and disruptive. To cope, you…*	8. Student Misbehavior. *A couple of students have been rude and disruptive. To cope, you…*	
command that they get back on task immediately; otherwise, there will be bad consequences. (dem)	demand that they return to their task immediately; otherwise, there will be serious consequences. (dem8)	A few words were changed
explain the reasons why you want them to behave properly. Later talk to them individually; you listen carefully to how they see things. (att)	explain why you want them to behave properly. Later you will talk to them individually and listen carefully to how they perceive things. (att8)	A few words were changed
communicate the classroom expectations for cooperation and prosocial skill. (clar)	communicate your expectations in terms of effort and attitude in class. (clar8)	A few words were changed
let it go, because it is too much of a pain to intervene. (aban)	are letting it go because it’s too compelling to intervene. (aban8)	A few words were changed
9. Practice Time. *It is time for students to practice what they have learned. You …*	9. Practice Time. *It is time for students to practice. You …*	
ask students which types of practice problems they may want to work on the most. (part)	suggest different levels of difficulty and ask the students at which level they would like to practice. (part9)	A few words were changed
demand that now is the time to work, whether they like it or not. Tell them that they sometimes need to learn to do things against their will. (dom)	demand that it’s time to work, whether they like it or not. You explain to them that sometimes they have to learn to do things against their will. (dom9)	A few words were changed
don’t plan too much and see how things evolve. (await)	don’t plan too much and watch how things develop. (await9)	A few words were changed
explain the solution to one problem step-by-step, then guide their progress and improvement on the follow-up problems. (clar)	set out step-by-step the key points that will guide their progress through the learning process. (guid9)	**Significant adaptation and modification of the strategy**
10. Arguing Students. *As the class ends, it comes to your attention that two students are arguing and offending each other. As the rest of the students leave the classroom, you ask the two students to remain so that you can…*	10. Arguing Students. *At the end of the lesson, you notice two students arguing and insulting each other. You…*	
take the arguing students aside: describe briefly what you saw and ask for their view and suggestions about what to do. (att)	ask both students to stay after class. You explain what you saw and ask them for their views on what solutions should be considered. (att10)	A few words were changed
be clear about what the classroom guidelines and expectations are. Indicate what helpful, cooperative behavior is. (clar)	clarify with these students your expectations and the desired attitude in class by taking them aside. (clar10)	A few words were changed
don’t intervene, just let students resolve things for themselves. (aban)	don’t interfere, you let the students sort it out amongst themselves. (aban10)	A few words were changed
tell them they should be ashamed of their behavior and that, if they continue, there will be sanction. (dom)	tell them that they should be ashamed of their behavior and that there will be a penalty if they continue. (dom10)	A few words were changed
11. Test Results. *You have finished scoring a test. Several students scored low again, even though you paid extra attention to this material last week. You…*	11. Evaluation Results. *You’ve just completed an evaluation. Several students did not pass, although you have paid particular attention to practicing these exercises in the last few lessons. You…*	
insist that low scores are unacceptable to you. Tell students that they must score higher for their own good. (dem)	insist that bad results are unacceptable to you. You tell students that they must do better next time. (dem11)	A few words were changed
help students revise their wrong answers so they understand what went wrong and how to improve. (guid)	help students understand why they did not succeed so that they understand what went wrong and how they can improve. (guid11)	A few words were changed
listen with patience and understanding to what the students say about the test performance. (att)	listen patiently and understandingly to what students have to say about their results. (att11)	A few words were changed
don’t spend class time on the low scoring students. (aban)	don’t spend time in class talking to students who have performed poorly. (aban11)	A few words were changed
12. Homework. *When assigning homework you …*	12. A student arrives several times late. *A student leaves the locker room late for the second time in a row. He/she seems to be somewhere else. You…*	
make it clear that the homework has to be done well; if not, bad consequences will follow. (dem)	**explain to the rest of the class that you are disappointed that he/she is late for the second time in a row. (dom12)**	**Significant adaptation and modification of the strategy**
communicate what it involves to competently do the homework. Check that everyone understands what is required to successfully accomplish the homework. (clar)	**repeat your expectations regarding punctuality in class. (clar12)**	**Significant adaptation**
offer a number of different homework exercises (e.g., three) and you ask students to pick a few of them (e.g., two). (part)	**take the student aside after the lesson and ask if anything is wrong. (att12)**	**Significant adaptation and modification of the strategy**
let the homework speak for itself rather than over-explaining everything. (aban)	**don’t say anything. At the end of the day, you can’t intervene with every student, you have to teach first. You focus on the lesson. (aban12)**	**Significant adaptation**

Note. Part = participative, att = attuning, guid = guiding, clar = clarifying, dem= demanding, dom = domineering, aban = abandoning, await = awaiting.

**Table 3:** Means, Reliabilities, and Correlations Among the Four Teaching Styles and Eight Identified Subareas in Belgium and France.

Dimension	N Items	M (SD)	ω	1	2	3	4	5	6	7	8	9	10	11
Teaching styles (N = 395)														
1. Autonomy support	12	4.48 (0.74)	0.76											
2. Structure	12	5.81 (0.60)	0.81	0.36 ***										
3. Control	12	3.07 (1.01)	0.86	−0.20 ***	0.05									
4. Chaos	12	2.31 (0.75)	0.77	−0.09	−0.28 ***	0.30 ***								
Subareas														
5. Participative	4	4.15 (1.01)	0.45	0.90 ***	0.16 **	−0.22 ***	0.01							
6. Attuning	8	5.49 (0.71)	0.74	0.79 ***	0.52 ***	−0.10 *	−0.17 ***	0.46 ***						
7. Guiding	7	5.84 (0.66)	0.79	0.46 ***	0.85 ***	−0.16 *	−0.24 ***	0.28 ***	0.56 ***					
8. Clarifying	5	5.76 (0.81)	0.69	0.11 *	0.79 ***	0.26 ***	−0.22 **	−0.04	0.28 ***	0.34 ***				
9. Demanding	7	3.44 (1.06)	0.79	−0.18 *	0.08	0.92 ***	0.22 ***	−0.21 ***	−0.10	−0.12 *	0.28 ***			
10. Domineering	5	2.53 (1.16)	0.76	−0.18 ***	−0.00	0.88 ***	0.33 ***	−0.18 ***	−0.12 *	−0.17 **	0.19 *	0.66 ***		
11. Abandoning	8	1.89 (0.64)	0.73	−0.15 **	−0.26 ***	0.42 ***	0.67 ***	−0.07	−0.22 **	−0.27 ***	−0.15 **	0.35 ***	0.42 ***	
12. Awaiting	4	2.71 (1.17)	0.72	−0.03	−0.22 ***	0.15 **	0.92 ***	0.03	−0.10 *	−0.16 ***	−0.20 ***	0.09	0.20 ***	0.32 ***

Notes. * *p* < 0.05; ** *p* < 00.01; *** *p* < 0.001

**Table 4:** Means, Reliabilities, and Correlations Among the Four Teaching Styles and Eight Identified Subareas in Belgium.

Dimension	N Items	M (SD)	ω	1	2	3	4	5	6	7	8	9	10	11	12
Teaching styles (N = 136)															
1. Autonomy support	12	4.93 (0.73)	0.80												
2. Structure	12	5.71 (0.54)	0.81	0.20 *											
3. Control	12	2.86 (0.88)	0.85	−0.33 ***	−0.08										
4. Chaos	12	2.44 (0.80)	0.84	−0.06	−0.32 ***	0.36 ***									
Subareas															
5. Participative	4	4.34 (1.03)	0.44	0.91 ***	0.00	−0.31 ***	0.00								
6. Attuning	8	5.52 (0.67)	0.78	0.78 ***	0.43 ***	−0.23 **	−0.14	0.46 ***							
7. Guiding	7	5.95 (0.56)	0.82	0.34 ***	0.78 ***	−0.18 *	−0.35 ***	0.14	0.53 ***						
8. Clarifying	5	5.39 (0.84)	0.82	−0.01	0.81 ***	0.30 ***	−0.17 **	−0.13	0.17 *	0.27 ***					
9. Demanding	7	3.24 (0.96)	0.79	−0.25 *	0.19 *	0.88 ***	0.18 *	−0.28 ***	−0.12	−0.08	0.32 ***				
10. Domineering	5	2.49 (1.02)	0.78	−0.32 ***	−0.03	0.90 ***	0.45 ***	−0.28 ***	−0.28 ***	−0.27 **	0.21 *	0.57 ***			
11. Abandoning	8	2.08 (0.69)	0.79	−0.14	−0.27 ***	0.41 ***	0.74 ***	−0.05	−0.23 **	−0.39 ***	−0.05	0.23 *	0.48 ***		
12. Awaiting	4	2.80 (1.17)	0.80	−0.01	−0.28 ***	0.25 **	0.92 ***	0.03	−0.06	−0.25 **	−0.20 *	0.11	0.32 **	0.43 ***	
Convergent validity (N = 69)															
13. Autonomy support ^a^	6	3.41 (0.69)	0.90	0.57 ***	−0.06	−0.12	0.19	0.55 ***	0.40 ***	0.17	−0.08	−0.15	−0.07	0.16	0.17
14. Structure ^a^	5	4.01 (0.48)	0.89	0.25 *	0.46 ***	−0.06	−0.02	0.09	0.40 ***	0.49 ***	0.25 *	0.04	−0.13	0.03	−0.04
15. Involvement ^a^	6	4.19 (0.45)	0.78	0.38 ***	0.27 *	−0.01	−0.00	0.24 *	0.47 ***	0.23 ^t^	0.20	−0.04	0.02	−0.02	0.02
16. Control ^b^	9	2.05 (0.49)	0.78	−0.07	−0.31 **	0.46 ***	0.23 ^t^	0.01	−0.18	−0.29 *	−0.20	0.28 *	0.50 ***	0.26 *	0.17

Notes. ^t^ *p* < 0.06; * *p* < 0.05; ** *p* < 0.01; *** *p* <.001; ^a^ Teacher as social context questionnaire, ^b^ Perceived Control teaching.

**Table 5:** Means, Reliabilities, and Correlations Among the Four Teaching Styles and Eight Identified Subareas in France (N = 259).

Dimension	N Items	M (SD)	ω	1	2	3	4	5	6	7	8	9	10	11	12
Teaching styles															
1. Autonomy support	12	4.76 (0.74)	0.76												
2. Structure	12	5.86 (0.62)	0.83	0.46 ***											
3. Control	12	3.18 (1.06)	0.86	−0.13 *	0.01										
4. Chaos	12	2.24 (0.71)	0.71	−0.13 *	−0.24 ***	0.31 ***									
Subareas															
5. Participative	4	4.05 (0.99)	0.41	0.90 ***	0.26 ***	−0.16 *	−0.05								
6. Attuning	8	5.47 (0.74)	0.75	0.80 ***	0.58 ***	−0.05	−0.20 ***	0.46 ***							
7. Guiding	7	5.79 (0.71)	0.80	0.50 ***	0.90 ***	−0.13 *	−0.22 ***	0.33 ***	0.57 ***						
8. Clarifying	5	5.96 (0.73)	0.72	0.25 ***	0.81 ***	0.19 **	−0.20 ***	0.18 **	0.39 ***	0.48 ***					
9. Demanding	7	3.55 (1.10)	0.80	−0.09	0.04	0.91 ***	0.22 ***	−0.12 *	−0.01	−0.11	0.20 ***				
10. Domineering	5	2.81 (1.20)	0.77	−0.14 *	−0.03	0.93 ***	0.35 ***	−0.17 *	−0.07	−0.12 *	0.14 *	0.69 ***			
11. Abandoning	8	1.80 (0.59)	69	−0.21 ***	−0.26 ***	0.50 ***	0.61 ***	−0.14 *	−0.23 ***	−0.27 ***	−0.10	0.45 ***	0.49 ***		
12. Awaiting	4	2.68 (1.17)	69	−0.05	−0.18 ***	0.12 *	0.92 ***	−0.02	−0.13 *	−0.13 *	−0.19 **	0.04	0.18 **	0.25 ***	
Antecedents															
13. Autonomous motivation	6	6.05 (0.70)	0.87	0.34 ***	0.40 ***	−0.11	−0.17 **	0.22 ***	0.39 ***	0.38 ***	0.30 ***	−0.08	−0.12 ^t^	−0.27 ***	−0.07
14. Controlled motivation	6	3.04 (0.47)	0.85	0.08	0.04	0.33 ***	0.07	0.02	0.14 *	−0.01	0.06	0.27 ***	0.34 ***	0.17 **	−0.00
15. Amotivation	3	1.60 (0.85)	0.65	−0.13 *	−0.07	0.27 ***	0.24 ***	−0.07	−0.16 *	−0.08	−0.02	0.19 **	0.30 ***	0.39 ***	0.09
16. Year of experience		20.94 (10.29)		0.09	0.16 **	0.03	0.09	0.08	0.07	0.11	0.17 **	0.01	0.04	−0.02	0.12

Notes.^t^ *p* < 0.06; * *p* < 0.05; ** *p* < 0.01; *** *p* < 0.001.

**Table 6:** Concurrent analysis with teachers’ motivation variables (N = 259).

	Participative	Attuning	Guiding	Clarifying	Demanding	Domineering	Abandoning	Awaiting	Autonomy Support	Structure	Control	Chaos
Gender	−0.07	−0.050	−0.08	−0.09	0.15 **	0.26 ***	0.18 **	0.13 *	−0.07	−0.10	0.22 ***	0.18 **
Year of experience	0.06	0.04	0.09	0.16 **	0.02	0.05	−0.02	0.12 ^t^	0.06	0.14 *	0.03	0.09
Autonomous motivation	0.22 **	0.36 ***	0.41 ***	0.32 ***	−0.05	−0.03	−0.13 *	−0.03	0.32 ***	0.43 ***	−0.04	−0.08
Controlled motivation	−0.01	0.11	−0.06	0.00	0.26 ***	0.30 ***	0.11 ^t^	−0.01	0.05	−0.04	0.30 ***	0.04
Amotivation	0.01	−0.03	0.10	0.10	0.11	0.21 ***	0.31 ***	0.07	−0.01	0.11	0.18 **	0.18 **
R^2^	0.06	0.17	0.17	0.13	0.12	0.24	0.21	0.04	0.13	0.20	0.21	0.10
∆R^2^	0.04 *	0.16 ***	0.14 ***	0.08 ***	0.09 ***	0.16 ***	0.17 ***	0.01	0.11 ***	0.15 ***	0.15 ***	0.05 **

Only results of regression of autonomous motivation, controlled motivation and amotivation on SIS dimensions and subareas are included. R^2^= total variance explained in teaching approach by control variables (Step 1) and main effects (Step 2). Δ*R*2 = additional variance explained in Step 2. ^t^ *p* < 0.06, * *p* < 0.05, ** *p* < 0.01, *** *p* < 0.001.

## Data Availability

The data are available on https://osf.io/fx53w/ (siscfabelfr); accessed on 7 July 2021.

## Supplementary Materials

The following are available online at https://www.mdpi.com/article/10.3390/ijerph18147342/s1, Table S1: French and Dutch translations.
