# Supplementary material for: Adopting the Situation in School Questionnaire to Examine Physical Education Teachers’ Motivating and Demotivating Styles Using a Circumplex Approach
Source: Int J Environ Res Public Health. 2021 Jul 9;18(14):7342. doi: 10.3390/ijerph18147342 (PMC8304182; doi:10.3390/ijerph18147342)
Supplement: Supplementary file 1 [file ijerph-18-07342-s001.zip › ijerph-1263227-supplementary.pdf]

Supplementary Material

Table S1. French and Dutch translation.

SIS-PE
1. Class Rules. At the beginning of the school year, you propose operating rules. You…<br>Règles de classe. Au début de l'année scolaire, vous proposez des règles de fonctionnement<br>Regels en richtlijnen. Aan het begin van het schooljaar voer je enkele regels en afspraken in. Hierbij…
…announce your expectations and the rules necessary for optimal cooperation. (clar1)
•Vous annoncez vos attentes et les règles nécessaires à une coopération optimale.
•maak je jouw normen en verwachtingen voor een goede samenwerking duidelijk.
…don't worry too much about the rules of operation and their application. You intervene when a problem arises. (await1)
•Vous ne vous préoccupez pas trop des règles de fonctionnement et de leur application. Vous intervenez quand un problème survient.
•ben je niet bekommerd om regels en afspraken. Je grijpt in wanneer een probleem zich stelt.
…set out the rules that students are expected to follow. You also list the penalties for breaking them. (dem1)
•Vous énoncez les règles que les élèves sont tenu-e-s de suivre. Vous énumérez également les sanctions en cas de leur non-respect.
•lijst je de regels en afspraken op die de leerlingen moeten volgen. Je somt ook de sancties op voor zij die niet gehoorzamen aan deze regels.
…invite students to suggest a few rules that will help them feel comfortable during the lesson. (part1)
•Vous invitez les élèves à proposer quelques règles qui les aideront à se sentir à l'aise durant la leçon.
•Vraag je aan de leerlingen om suggesties te doen voor regels en richtlijnen, zodat ze zich op hun gemak voelen tijdens de les.
2. Lesson Plan. In preparing for your class, you develop a lesson plan. Your priority is to...<br>Début de la leçon. La leçon commence, votre priorité est de<br>Lesvoorbereiding. Je maakt een lesvoorbereiding. Je prioriteit is…
…offer challenges to the best students and provide sufficient support to exceptional students throughout their learning. (guid2)
•Proposer des défis aux meilleur-e-s élèves et apporter suffisamment d'aide aux élèves en difficulté tout au long de leur apprentissage.
•sterke leerlingen voldoende uitdaging te bieden en minder sterke leerlingen voldoende hulp te bieden in hun leerproces.
…don't plan the lesson too much. It will unfold on its own. (await2)
•Ne pas trop planifier la leçon. Elle se déroulera d'elle-même.
•niet te veel te plannen en te zien hoe de les verloopt.

…propose exercises that are pleasant, interesting, or very attractive. (att2)
•Proposer des exercices plaisants, intéressants, ou très attrayants.
•oefeningen te zoeken die de leerlingen leuk, interessant of boeiend vinden.
…propose a lesson plan for all students to follow. There are no exceptions or excuses. (dem2)
•Proposer un plan de leçon que tous les élèves doivent suivre. Il n'y a ni exceptions, ni excuses.
•een opbouw te voorzien die alle leerlingen verplicht moeten volgen. Er zijn geen uitzonderingen, geen excuses.

3. Starting Class. The class period begins. You…<br>Début de la leçon. La leçon commence.<br>De les begint. De les begint. Jij …

set up a clear and easy-to-follow organization. (clar3)
•Vous mettez en place une organisation claire et facile suivre.
•zorgt voor een duidelijke en gemakkelijk te volgen indeling.
start the lesson and let it unfold. (await3)
•Vous commencez la leçon et laissez les choses se dérouler.
•start de les en je neemt vervolgens de dingen zoals ze komen.
insist strongly that students must put into practice what is taught. Your duty is to teach, their duty is to learn. (dem3)
•Vous insistez fermement sur le fait que les élèves doivent mettre en pratique ce qui est enseigné. Votre devoir est d'enseigner, leur devoir est d'apprendre.
•staat er sterk op dat de leerlingen moeten oefenen wat ze aangeleerd krijgen. Het is jouw plicht om les te geven en hun plicht om te leren.
are interested in what students know about the learning theme. (att3)
•Vous êtes intéressé-e de savoir ce que les élèves connaissent à propos du thème d'apprentissage.
•bent geïnteresseerd om te horen wat de leerlingen weten over het lesonderwerp.

4. Motivating Students. You would like to motivate students during class. You decide to…<br>Motiver les élèves. Vous voulez motiver vos élèves durant les cours.<br>Leerlingen motiveren. Jij wilt de leerlingen motiveren tijdens de les LO. Jij …

..don't take care of unmotivated students, you don't manage to improve their motivation. (aban4)
•Vous ne vous occupez pas des élèves non motivé-e-s, vous n'arrivez pas à améliorer leur motivation.
•laat de ongemotiveerde leerlingen gewoon hun gang gaan; omdat je hun motivatie toch niet kan verbeteren.

…whistle and say loud and clear, "Now let's focus and get busy." (dem4)
•Vous sifflez et dites haut et fort : “Maintenant, on se concentre et on s’active”.
•geeft een fluitsignaal en roept luid: “Nu is het tijd om geconcentreerd en actief aan de slag te gaan!”
…give positive feedback, while offering help and advice when necessary. (guid4)
•Vous donnez des retours positifs, tout en proposant, lorsque c’est nécessaire, de l’aide et des conseils.
•geeft positieve feedback, en biedt waar nodig hulp en begeleiding.
…offer students a number of different activities that they can choose for the next cycle of education. (part4)
•Vous proposez aux élèves un certain nombre d’activités différentes qu’ils/elles peuvent choisir pour le prochain cycle d’enseignement.
•je biedt de leerlingen enkele mogelijke lesonderwerpen voor de komende periode aan waaruit ze kunnen kiezen.

5. Students Complain. During a difficult exercise in the lesson, students start to complain. In response, you…
Plaintes des élèves. Pendant un exercice difficile de la leçon, les élèves commencent à se plaindre
Leerlingen klagen. Tijdens een moeilijkere oefening in de les merk je dat enkele leerlingen beginnen te klagen. Jij …

…consider their frustration and explain the importance of this exercise. (att5)
•Vous prenez en considération leur frustration et expliquez l’importance de cet exercice.
•erkent hun frustratie en je licht het belang van de oefening toe.
…insist that they keep focusing. They must learn these exercises for their own good. (dem5)
•Vous insistez pour qu’ils/elles continuent à se concentrer. Ils/elles doivent apprendre ces exercices pour leur bien.
•staat er op dat de leerlingen geconcentreerd verder werken. Ze moeten de oefeningen uitvoeren omwille van hun eigen bestwil.
…show them the exercise step-by-step and teach them a strategy that helps them pass the exercise. (guid5)
•Vous montrez l’exercice pas-à-pas et leur enseignez une stratégie qui les aide à réussir l'exercice.
•overloopt de oefening stap voor stap en brengt een behulpzame strategie bij om de oefening uit te voeren.
…ignore the wailing and the complaining. They must learn to overcome obstacles on their own. (aban5)
•Vous ignorez les lamentations et les plaintes. Ils/elles doivent apprendre à dépasser les obstacles par eux-mêmes.
•negeert het zeuren en klagen. Ze moeten zelf leren hoe ze obstakels kunnen overwinnen.

6. Needing Extra Effort. You are presenting a difficult exercise that requires a lot of effort for the students. In doing so, you…
Efforts supplémentaires nécessaires. Vous présentez un exercice difficile qui requiert beaucoup d'efforts pour les élèves
Een extra inspanning is nodig. Je geeft een zware en lastige oefening die veel inspanning van de leerlingen vraagt. Je…

…are not too worried, because students need to understand for themselves how much effort they have to put in. (aban6)

•Vous n’êtes pas trop inquiet-e, car les élèves doivent comprendre par eux-mêmes l'effort qu'ils/elles doivent fournir.
•maakt je niet te veel zorgen. De leerlingen moeten voor zichzelf uitmaken hoeveel inspanning ze leveren.
…try to find new ways to make the exercise more fun and interesting for the students. (att6)
•Vous essayez de trouver des nouveaux moyens pour rendre l’exercice plus plaisant et intéressant pour les élèves.
•zoekt nieuwe manieren om de oefening leuker en interessanter te maken voor de leerlingen.
…firmly insist that "playtime" is over and that now they must show what they are worth. (dom6)
•Vous insistez fermement que la « récréation » est finie et que maintenant ils/elles doivent montrer ce qu’ils/elles valent.
•beveelt hen dat de speeltijd voorbij is en dat ze nu kunnen bewijzen wat ze waard zijn.
…help the students with concrete advice on how to do the exercise successfully. (guid6)
•Vous aidez les élèves avec des conseils concrets sur la manière de réussir l’exercice.
•maakt de leerling met concrete tips duidelijk hoe ze de oefening tot een goed einde kunnen brengen.

7. Anxiety Surfaces. During an exercise, you notice that some students show signs of anxiety. Sensing that anxiety, you…
Signes d’anxiété. Durant un exercice, vous remarquez que certain-e-s élèves démontrent des signes d’anxiété. Ressentant cette anxiété, …
Leerlingen vertonen angst. Tijdens een bepaalde oefening merk je dat sommige leerlingen schrik hebben. Wanneer je hun schrik opmerkt, dan…

…talk to the students and suggest that they engage in another exercise that scares them less or not at all. (part7)
•Vous parlez aux élèves et leur proposer de s’engager dans un autre exercice qui les effraie moins ou pas du tout.
•spreek je de leerlingen aan en vraag je hen om samen met jou een vervangoefening te bedenken waarbij ze geen/minder schrik hebben.
…insist that they need to move beyond this state and act in a more mature way. (dom7)
•Vous insistez sur le fait qu’ils/elles doivent dépasser cet état et agir de manière plus mature.
•sta je er op dat ze zich erover zetten en dat dat ze zich op een meer volwassen wijze beginnen te gedragen.
…try to reduce their anxiety by breaking down the …steps needed to complete the exercise so that they feel able to do it successfully. (guid7)
•Vous essayez de diminuer leur anxiété en décomposant les étapes nécessaires pour réaliser l’exercice, de façon à ce qu’ils/elles se sentent capables de le réussir.
•probeer je de schrik weg te nemen door tussenstappen aan te bieden die nodig zijn voor de leerlingen om zich bekwaam te voelen.
…don't have to worry about their anxiety, it will pass on by itself. (aban7)
•Vous ne vous inquiétez pas de leur anxiété, cela passera tout seul.
•maak je jou geen zorgen over de angst. Deze zal wel vanzelf overgaan.

8. Student Misbehavior. A couple of students have been rude and disruptive. To cope, you…<br>Comportements perturbateurs des élèves. Certain-e-s élèves ont été impoli-e-s et perturbateur-trices<br>Wangedrag bij leerlingen. Een aantal leerlingen hebben zich onbeleefd en storend gedragen. Jij…
…demand that they return to their task immediately; otherwise, there will be serious consequences. (dem8)<br>•Vous exigez qu'ils/elles retournent à leur tâche immédiatement ; sans quoi, il y aura de lourdes conséquences.<br>•beveelt hen dat ze meteen met hun oefening dienen verder te gaan, anders zullen er nare gevolgen zijn.<br>…explain why you want them to behave properly. Later you will talk to them individually and listen carefully to how they perceive things. (att8)<br>•Vous expliquez les raisons pour lesquelles vous voulez qu'ils/elles se comportent correctement. Plus tard, vous leur parlerez individuellement et écouterez attentivement comment ils/elles perçoivent les choses.<br>•legt hen uit waarom je verwacht dat ze zich goed gedragen. Later praat je met hen individueel; je luistert aandachtig naar hoe zij de zaken zien.<br>…communicate your expectations in terms of effort and attitude in class. (clar8)<br>•Vous leur communiquez vos attentes en matière d'effort et d'attitude en classe.<br>•geeft aan wat jij verwacht op het vlak van inzet en attitude in de les.<br>…are letting it go because it's too compelling to intervene. (aban8)<br>•Vous laissez aller, car c'est trop contraignant d'intervenir.<br>•laat het betijen omdat het teveel moeite kost tussen te komen.
9. Practice Time. It is time for students to practice. You …<br>Pratique. Il est temps pour les élèves de s'exercer.<br>Oefentijd. Het ogenblik is aangebroken voor de leerlingen om een beweging in te oefenen. Je…
…suggest different levels of difficulty and ask the students at which level they would like to practice. (part9)<br>•Vous proposez différents niveaux de difficulté et demandez aux élèves à quel niveau ils/elles aimeraient s'exercer.<br>•voorziet verschillende niveaus en vraagt aan de leerlingen op welk niveau ze willen oefenen.<br>…demand that it's time to work, whether they like it or not. You explain to them that sometimes they have to learn to do things against their will. (dom9)<br>•Vous exigez qu'il est temps de travailler, que cela leur plaise ou non. Vous leur expliquez que parfois qu'ils/elles doivent apprendre à faire des choses contre leur gré.

•beveelt hen dat het nu tijd is om te werken, of ze dit nu leuk vinden of niet. Je zegt hen dat ze nu eenmaal soms iets tegen hun zin moeten leren doen.
…don't plan too much and watch how things develop. (await9)
•Vous ne planifiez pas trop et regardez comment évoluent les choses.
•plant niet te veel en wacht af hoe de zaken evolueren.
… set out step-by-step the key points that will guide their progress through the learning process. (guid9)
•Vous énoncez étape par étape les points clés qui guideront leurs progrès au cours du processus d'apprentissage.
•geeft stap voor stap de aandachtspunten mee en begeleidt de leerlingen bij hun vooruitgang in hun leerproces.

10. Arguing Students. At the end of the lesson, you notice two students arguing and insulting each other. You…
Disputes entre élèves. A la fin de la leçon, vous remarquez que deux élèves se disputent et s'insultent.
Ruziënde leerlingen. Aan het einde van de les merk je dat twee leerlingen ruzie aan het maken zijn en elkaar beledigen. Je…

…ask both students to stay after class. You explain what you saw and ask them for their views on what solutions should be considered. (att10)
•Vous demandez aux deux élèves de rester après le cours. Vous leur expliquez ce que vous avez vu et leur demandez leur point de vue quant aux solutions à envisager.
•Vraagt de ruziemakers om na te blijven. Je legt hen uit wat je gezien hebt en je vraagt naar hun mening over hoe dit kan opgelost worden.
…clarify with these students your expectations and the desired attitude in class by taking them aside. (clar10)
•Vous clarifiez auprès de ces élèves vos attentes et l'attitude souhaitée en cours, en les prenant à part.
•roept de ruziemakers bij je om duidelijk te maken wat je verwachtingen zijn en wat een positieve attitude in de les LO inhoudt.
…don't interfere, you let the students sort it out amongst themselves. (aban10)
•Vous n'intervenez pas, vous laissez les élèves régler cela entre eux.
•komt niet tussen. Je laat de leerlingen zelf hun problemen oplossen.
…tell them that they should be ashamed of their behavior and that there will be a penalty if they continue. (dom10)
•Vous leur dites qu'ils/elles devraient avoir honte de leur comportement et qu'il y aura une sanction s'ils/elles continuent.
•maakt de ruziemakers duidelijk dat ze beschaamd moeten zijn voor hun gedrag en dat wanneer ze zo verder gaan, er sancties zullen volgen.

11. Evaluation Results. You've just completed an evaluation. Several students did not pass, although you have paid particular attention to practicing these exercises in the last few lessons. You…
Résultats d'évaluation. Vous venez de terminer une évaluation. Plusieurs élèves n'ont pas réussi, bien que vous ayez accordé une attention particulière à la pratique de ces exercices durant les dernières leçons.

Resultaten na een evaluatie. Je bent net klaar met een evaluatie. Verschillende leerlingen scoren alweer laag ondanks de extra aandacht die je vorige lessen gaf aan deze leerlingen. Je…
…insist that bad results are unacceptable to you. You tell students that they must do better next time. (dem11)<br>•Vous insistez sur le fait que de mauvais résultats sont inacceptables pour vous. Vous dites aux élèves qu'ils/elles doivent faire mieux la prochaine fois.<br>•wijst erop dat lage scores voor jou niet aanvaardbaar zijn. Je zegt dat ze de volgende keer omwille van hun eigen bestwil beter moeten presteren<br>…help students understand why they did not succeed so that they understand what went wrong and how they can improve. (guid11)<br>•Vous aidez les élèves à comprendre pourquoi ils/elles n'ont pas réussi afin qu'ils comprennent ce qui s'est mal passé et comment ils/elles peuvent s'améliorer.<br>•helpt de leerlingen inzicht krijgen in de oorzaken van de lage scores zodat ze begrijpen wat er fout ging en weten hoe ze kunnen verbeteren.<br>…listen patiently and understandingly to what students have to say about their results. (att11)<br>•Vous écoutez patiemment et de manière compréhensive ce que les élèves ont à dire sur leurs résultats.<br>•je luistert geduldig en met begrip naar wat de leerlingen zelf zeggen over hun resultaten.<br>…don't spend time in class talking to students who have performed poorly. (aban11)<br>•Vous ne passez pas du temps en classe à discuter avec les élèves qui ont obtenu de mauvais résultats.<br>•verspilt tijdens de les geen tijd meer aan de bespreking van de evaluaties van de leerlingen die slecht scoren.
12. A student arrives several times late. A student leaves the locker room late for the second time in a row. He/she seems to be somewhere else. You…<br>Un-e élève arrive plusieurs fois en retard. Un-e élève sort du vestiaire en retard pour la deuxième fois consécutive. Il/elle a l'air ailleurs. Que faites-vous ?<br>Een leerling komt meermaals te laat. Een leerling is nu al voor de tweede les op rij te laat uit de kleedkamer en maakt een afwezige indruk. Wat doe je? Je…
…explain to the rest of the class that you are disappointed that he/she is late for the second time in a row. (dom12)<br>•Vous expliquez au reste de la classe que vous êtes déçu-e qu'il/elle soit en retard pour la seconde fois consécutive.<br>•maakt voor de ganse klas duidelijk dat je ontgoocheld bent in hem/haar, want het is nu al de tweede keer op rij.<br>…repeat your expectations regarding punctuality in class. (clar12)<br>•Vous répétez vos attentes concernant la ponctualité en classe.

•herhaalt de verwachtingen over op tijd komen in de les.
…take the student aside after the lesson and ask if anything is wrong. (att12)
•Vous prenez l'élève à part après la leçon et vous lui demandez si quelque chose ne va pas.
•neemt de leerling na de les apart en vraagt of er hem/haar iets dwarszit.
…don't say anything. At the end of the day, you can't intervene with every student, you have to teach first. You focus on the lesson. (aban12)
•Vous ne dites rien. En fin de compte, vous ne pouvez pas intervenir auprès de chaque élève, vous devez avant tout enseigner. Vous vous concentrez sur la leçon.
•zegt er niets over; uiteindelijk kan je je niet met elke leerling inlaten, want je hebt ook nog les te geven. Je focust je op jouw les.

Note. Part = participative, att = attuning, guid = guiding, clar = clarifying, dem= demanding, dom = domineering, aban = abandoning, await = awaiting

To calculate the scores of each dimension:

Autonomy support = mean of participative (part1) (part4) (part7) (part9) and attuning (att2) (att3) (att5) (att6) (att8) (att10) (att11) (att12) items

Structure = mean of guiding (guid2) (guid4) (guid5) (guid6) (guid7) (guid9) (guid11) and clarifying (clar1) (clar3) (clar8) (clar10) (clar12) items

Control = mean of demanding (dem1) (dem2) (dem3) (dem4) (dem5) (dem8) (dem11) and domineering (dom6) (dom7) (dom9) (dom10) (dom12) items

Chaos = mean of abandoning (aban4) (aban5) (aban6) (aban7) (aban8) (aban10) (aban11) (aban12) and awaiting (await1) (await2) (await3) (await9) items
